# Multiplex PCR based screening for microdeletions in azoospermia factor region of Y chromosome in azoospermic and severe oligozoospermic south Indian men

**Published:** 2013-03

**Authors:** Ramaswamy Suganthi, VV Vijesh, Sanjay Jayachandran, Jahangir Ali Fathima Benazir

**Affiliations:** *Department of Biotechnology, Dr. G. R. Damodaran College of Sciences, Coimbatore, 641014, India.*

**Keywords:** *Y chromosomal microdeletions*, *AZF region*, *Male infertility*, *Multiplex PCR*

## Abstract

**Background:** Y chromosomal microdeletion is an important genetic disorder, which may arise due to intrachromosomal recombination between homologous sequences in the male specific region of the human Y chromosome. It is frequently associated with the quantitative reduction of sperm. The screening for Y chromosomal microdeletions has a great clinical value.

**Objective:** To develop a sequence tagged site (STS) based multiplex PCR protocol, which could be specific for the rapid detection of AZF deletions and thereby estimating the frequency of AZF sub deletions in infertile South Indian men.

**Materials and Methods:** In the current study, PCR based Y chromosomal microdeletion screening analysis was performed in 75 men including 30 non-obstructive azoospermic men, 20 severe oligozoospermic, and 25 normozoospermic fertile men (controls) using 15 known STS primer pairs mapped within the AZF locus. Deletion frequency was estimated after successful PCR amplification.

**Results: **We designed and optimized a STS based multiplex PCR protocol, which could be helpful for the clinicians to detect the Y chromosomal deletions rapidly and specifically. In our study, we estimated an overall deletion frequency of 36%. Among these 12 (40%) were azoospermic and 6 (30%) were oligozoospermic. No microdeletions were observed in normozoospermic fertile men.

**Conclusion: **Our Study emphasizes the fact that Y chromosomal microdeletion screening tests are unavoidable in the workup of idiopathic male infertility. Mandatory screening for Y deletions should be done in all azoospermic and severe oligozoospermic patients before undergoing assisted reproductive technology.

## Introduction

Infertility silently emerges as a major public problem related to reproductive health. It adversely affects the social, psychological and economic status of the affected individuals and couples. It is estimated that the global prevalence of infertility ranges from 3.5-16.7% in developed nations and from 6.9-9.3% in developing and under developed nations (1). 

Astonishingly, a male factor can be observed in about half of the cases (2). Even though there were several proposed causes of male infertility, the causative factor of 60-75% cases are still unknown and termed as idiopathic male infertility (3). In these cases, it is speculated that the genetic factors may directly or with the interaction of environmental factors act at some stages of testicular development and disrupt spermatogenesis (4, 5). Disruption in spermatogenesis will result in qualitative decline and quantitative reduction of sperm. Azoospermia and oligozoospermia are frequently observed in men with impaired spermatogenesis, and most of these patients seek for intracytoplasmic sperm injection (ICSI) (6).

ICSI is a revolutionized assisted reproductive technique (ART) which fulfills the dream of millions to have their own baby (7, 8). Since the ICSI, technique bypasses all the natural barriers related to natural fertilization, it raises serious concern about the transmission of known and unknown genetic defects including Y chromosomal microdeletions to the descendants (9-13). This implies the necessity for testing genetic defects in infertile men before undergoing ICSI.

Y chromosomal microdeletion is defined as the missing up of gene or gene sequences from the functionally active part of the Y chromosome. It represents the most common structural chromosomal anomaly associated with non-obstructive azoospermia and severe oligozoospermia (14). To date, most of the studies on Y deletions are centred on a hotspot region known as the azoospermia factor (AZF), which is located at the distal portion of the long arm of the Y chromosome (Yq11.2) (15). 

Later on, Vogt *et al* reclassified the concept of the azoospermia factors into three non-overlapping regions, AZFa, AZFb and AZFc (16). Recent evidence directs to the existence of new pattern of AZF deletions in which distal part of AZFb overlaps with the proximal region of AZFc (17). Each region contains candidate genes, which are essential for normal development of germ cells. So far, at least fourteen proteins encoding Y genes were identified in AZF locus (18). 

The homologous recombination between identical Y-specific repeats generates deletions that cause the removal of functional AZF genes which in turn leads to spermatogenic failure (19). In the current study, we made an effort to develop a STS based multiplex PCR protocol to determine the frequency of Y chromosomal microdeletions in azoospermic and severe oligozoospermic infertile South Indian men.

## Materials and methods

**Patient selection**

The study was carried out on non-obstructive azoospermic and severe oligozoospermic infertile men referred to leading ART centres in South India. Patient selection was done based on the alterations detected in sperm count according to WHO guidelines (20). We excluded the patients with known causes of infertility like, karyotype abnormalities, obstructive azoospermia, varicocele and testicular tumors. 

A total of 75 men, including 30 non-obstructive azoospermic men, 20 severe oligozoospermic and 25 normozoospermic fertile men (Controls) were enrolled in the study. Written informed consent was obtained from all the patients enrolled in the study. One drop of blood embedded in Whatmann filter paper in sterile eppendorf were collected and transported to the laboratory in airtight cool packs and stored in the refrigerator for screening.

**DNA Isolation**

Genomic DNA was extracted from a single drop of peripheral blood samples using a rapid DNA extraction protocol (21). The concentrated DNA was diluted using RNAse free water (1:3 ratios) before being analysed by multiplex PCR.

**Multiplex PCR screening analysis**

The processed DNA of patients was subjected to multiplex PCR using 15 sets (Five multiplex set) of STS (Table I). Each primer pair amplifies a specific region of the AZF locus located in the long arm of Y chromosome. The AZF specific STS primers were selected according to the guidelines jointly recommended by the European Academy of Andrology and the European Molecular Genetics Quality Network and also from previous literatures (22-24).

The multiplex PCR reaction comprised a total volume of 16µl of which 8µl was the master mix (Qiagen, Germany), 5µl of multiplex STS primer mix and 3µl of diluted DNA. Amplifications were carried out on eppendorf thermocycler.40 cycles were performed as follows: 94^o^C for 5 min for denaturation, 55^o^C for 45 sec for primer annealing and 67^o^C for 4 min for extension. 

The programs were followed by the final extension step at 67^o^C for 4 min. The PCR products were then analyzed by electrophoresis at 50 V on 1.5% agarose gels (Himedia, Mumbai) and visualized by staining the gel with ethidium bromide (10.5µg/ml). Failure of amplification of any STS markers indicated microdeletion by comparing it with the control. Sample showing deletions were tested at least three times with single primer to confirm the results.

**Statistical analysis**

The frequency distribution of Y chromosomal microdeletions was calculated using Microsoft Excel 2010, Windows Installer version.

## Results

**Figure 1:**
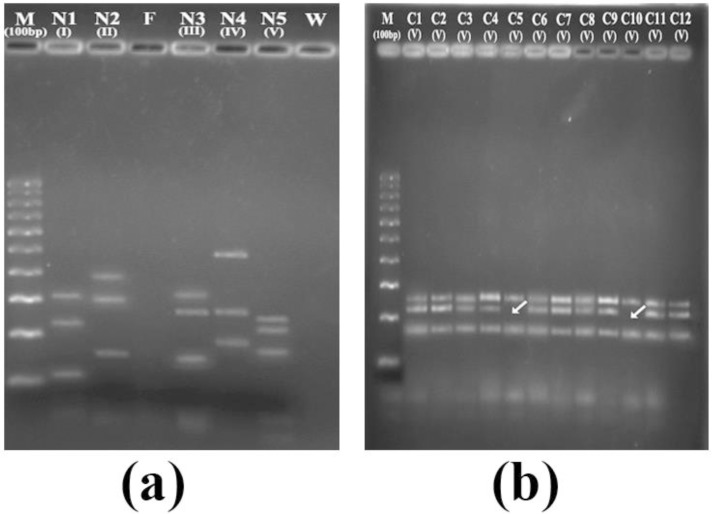
Gel images showing the multiplex PCR products.

Thirty non-obstructive azoospermic, twenty severe oligozoospermic infertile men and twenty five normozoospermic fertile men (Controls) were screened for Y chromosomal microdeletions using multiplex PCR method. DNA was separated from the peripheral blood leucocytes of all patients, and PCR amplification was successfully performed using AZF specific STS markers. PCR amplification produced bands of expected size for all the fifteen AZF loci investigated in all fertile controls (Figure 1). 

**Figure 2:**
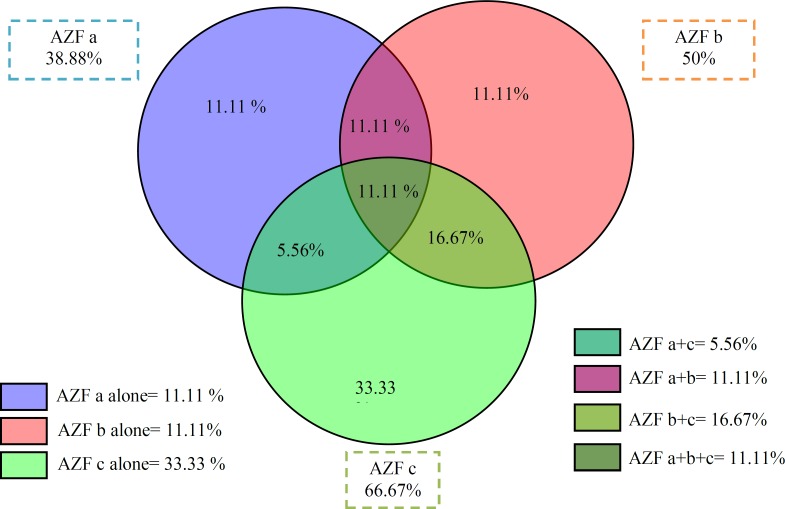
Vendiagram showing AZF deletions in the human Y chromosome.

Eighteen patients, including twelve azoospermic and six oligozoospermic patients showed deletion of one or more STS markers corresponding to AZF locus. The details of the results are shown in Figure 2 and summarized in Table II.

**Table I:** Sequence-Tagged Sites (STS) primer used for Y chromosomal microdeletion analysis

**Multiplex primer sets**	**STS**		**Locus**	**Primer sequence**	**Base pairs (bp)**
Multiplex primer set I					
	DFFRY		AZFa		111
				F:5’GAGCCCATCTTTGTCAGTTTA C 3’	
				R: 5’CTGCCAATTTTCCACATCAAC C 3’	
	sY143		AZFb		311
				F: 5’GCAGGATGAGAAGCAGGTAG 3’	
				R: 5’CCGTGTGCTGGAGACTAATC 3’	
	sY158		AZFc		231
				F:5’CTCAGAAGTCCTCCTAATAGTTC 3’	
				R: 5’ACAGTG GTT TGT AGC GGG TA 3’	
Multiplex primer set II					
	DBY		AZFa		169
				F: 5’AGTTTATTCTAACCTAGG CAAACG 3’	
				R: 5’TCCAACCAGGCCTGTAGTGAGGCC 3’	
	sY134		AZFb		301
				F: 5’GTCTGCCTCACCATAAAACG 3’	
				R: 5’ACCACTGCCAAAACTTTCAA 3’	
	sY254		AZFc		400
				F: 5’GGGTGTTACCAGAAG GCAAAA 3’	
				R: 5’ GAACCGTATCTACCAAAG CAG C 3’	
Multiplex primer set III					
	sY84	AZFa			320
				F: 5’AGAAGGGTCTGAAAGCAG GT 3’	
				R: 5’GCCTACTACCTGGAGGCTTC 3’	
	sY127	AZFb			274
				F: 5’GGCTCACAAACGAAAAGAAAA 3’	
				R: 5’CTGCAGGCAGTAATAAGGGA 3’	
	CDY	AZFc			132
				F: 5’TCATACATTCCAATTGTACTG G 3’	
				R: 5’TTCTATCCCTCGGGCTGAGCTC 3’	
Multiplex primer set IV					
	sY83		AZFa		275
				F: 5’CTTGAATCAAAGAAGGCCCT 3’	
				R: 5’CAATTTGGT TTGGCTGACAT 3’	
	sY143		AZFb		196
				F: 5’AGCTTCTAT TCGAGGGCT TC 3’	
				R: 5’CTCTCTGCAATCCCTGAC AT 3’	
	sY283		AZFc		497
				F: 5’CAGTGATACACTCGGACTTGTGTA 3’	
				R: 5’GTTATTTGAAAAGCTACACGG G 3’	
Multiplex primer set V					
	sY90		AZFa		176
				F: 5’CAGTGCCCCATAACACTTTC 3’	
				R: 5’ATGGTAATACAGCAGCTCGC 3’	
	sY117		AZFb		260
				F: 5’GTTGGTTCCATGCTCCATAC 3’	
				R: 5’CAGGGAGAGAGC CTTTTACC 3’	
	sY158		AZFc		231
				F: 5’ CTCAGAAGT CCTCCTAATAGTT 3^’^	
				R: 5’ACAGTGGTTTGTAGCGGGTA 3’	

**Table II:** Frequencies of AZF microdeletions

**Spermatogenetic defect (Spermatozoa in ejaculate)**	**Total patients screened**	**Sample studied**	**No. of STS markers used**	**Karyotype**	**Patients with** **microdeletions**	**Frequency of microdeletions (%)**
Non-obstructive Azoospermia (None)	30	Blood	15	normal*	12	40%
Oligozoospermia (˂5 mln/ml)	20	Blood	15	“	6	30%
Normozoospermia (˃20 mln/ml)	25	Blood	15	“	0	-
Total	75	-	15	-	18	36%

* All patients have normal karyotype

(a) Amplified multiplex PCR bands of normozoospermic fertile men (control) using various multiplex primer set. Lane 1, 100bp molecular weight marker , Lane 2,3,5,6,7 showing PCR amplified products using multiplex primer set I, II,III,IV & V respectively, Lane 4, female DNA, Lane 8, water. (b) Amplified multiplex PCR bands of infertile clinical samples using multiplex primer set V. The arrow marks indicates the deletion in corresponding STS marker.

## Discussion

A gene cluster which is essential for the normal spermatogenesis is located on interval 6 of the long arm of the Y chromosome (i.e., Azoospermia factor) (15, 16). Deletions in AZF locus are the most common event seen in azoospermic and severe oligozoospermic patients (14). Worldwide the incidence of AZF deletions reported in infertile samples is about 1-55.5% and none of the deletions were observed in a large number of fertile controls (25-27). 

The average frequency of Y chromosomal microdeletions in Indian studies comes around 7% (28). The lack of stringent patient selection criteria, the difference in experimental design and ethnic variations among different populations accounts for the variation in frequency (29, 30). In the present study, we observed deletions in 18 patients with a frequency range of 36%. Among these 12 (40%) were azoospermic and 6 (30%) were oligozoospermic, and our result are in accordance with the previous literatures. The small sample size (75 patients) and stringent patient inclusion criteria may accounts for the slight increase in the deletion frequency. 

Furthermore, Y chromosome microdeletions in azoospermic patients were found to be at a much higher rate than the oligozoospermic patients (31). Our study also reflect this finding and strengthen the speculation that Y chromosome microdeletions are associated with the quantitative reduction than the qualitative decline of sperm (30). 

The presence of the large number of repetitive sequence and massive palindromes in the male specific region of Y chromosomes accelerates the intrachromosomal recombination between homologous sequences, which in turn remove the AZF genes partially or completely (19). The removal of candidate genes from the AZF locus has variable clinical consequences. AZFc types of deletions are most frequently observed in a majority of the Y chromosomal microdeletion screening studies. This may be due to the presence of a large number of repetitive sequences compared to another locus (32). 

It is also speculated that there is a positive correlation between the size of the targets available for homologous recombination and incidence of each deletion (33). AZFc (66.67%) was the most frequently deleted region found in our study followed by AZFb (50%) and AZFa (38.88%).The relative frequency of AZFa, b and c region in infertile men in this study is AZFa alone in (11.11%); AZFb alone in (11.11%); AZFc alone in (33.33%); AZFa+c (5.56%), AZFa+b (11.11%), AZFb+c(16.67%) and AZFa+b+c deletions detected in two people (11.11%) (Figure 2). Hence the significant frequency rate of AZF deletions indicates the growing need of Y chromosomal screening analysis in infertile patients. 

AZFa deletions are responsible for the spermatogenic disruption, which leads to Sertoli cell-only (SCO) syndrome. A complete deletion of AZFa locus removes 792kb, including the two candidate genes *USP9Y* and *DBY* (34). AZFb deletions are associated with complete absence of elongating spermatids and spermatozoa or SCOS (29, 35, 36). AZFc deletions quantitatively reduce sperm density and show capricious phenotypical features ranges from azoospermia to mild/severe oligozoospermia (32). 

Furthermore, the retrieval of mature sperm upon testicular sperm extraction (TESE) for the use in IVF/ICSI is practically impossible in AZFa and AZFb deleted patients whereas sperm retrieval was possible in AZFc deleted men. At the same time risk associated with transmission of AZFc deletions was also reported (36-39) Hence the Y chromosomal microdeletion screening itself has a diagnostic, prognostic and a preventive value. The Y chromosomal microdeletion tests are now a reality in most of the andrology labs. However, the major hurdle faced by the Y chromosomal microdeletion screening is the lack of well standardized protocols. PCR amplification using STSs has most commonly used to identify microdeletions in the Y-chromosome. The variation in the number of STS markers used in various studies also made confusion. Now it is clear that there is no correlation between the number of STS markers used and the deletion frequency. 

The usage of a minimum number of highly specific, non-polymorphic STS markers will protect against inaccuracy (9). The simultaneous amplification of several loci in one reaction is achieved with the help of multiplex PCR. The optimization of multiplex PCR often poses difficulties in making different primer pairs work together. In the present study, we successfully develop a multiplex PCR protocol with 15 sets of AZF specific STS markers. The multiplex PCR protocol we developed is convenient, cost effective, fewer time-consuming and specific for the rapid detection of AZF deletions in infertile patients.

## Conclusion

Bypassing the barriers of natural fertilization with the help of ART without proper screening analysis may increase the number of infertile population. New innovations and researches are essential for identifying known, and unknown molecular genetic defects associated with ART. Here we presented a simple multiplex protocol for detecting AZF deletions in azoospermic and severe oligozoospermic South Indian men before undergoing ART.
